# Biofilm-Induced Antibiotic Resistance in Clinical *Acinetobacter baumannii* Isolates

**DOI:** 10.3390/antibiotics9110817

**Published:** 2020-11-17

**Authors:** Abebe Mekuria Shenkutie, Mian Zhi Yao, Gilman Kit-hang Siu, Barry Kin Chung Wong, Polly Hang-mei Leung

**Affiliations:** 1 Department of Health Technology and Informatics, the Hong Kong Polytechnic University, Hong Kong, China; abebe-mekuria.shenkutie@connect.polyu.hk (A.M.S.); mian-zhi.yao@connect.polyu.hk (M.Z.Y.); gilman.siu@polyu.edu.hk (G.K.-h.S.); 2 Department of Pathology, United Christian Hospital, Hong Kong, China; wongkc@ha.org.hk

**Keywords:** *Acinetobacter baumannii*, biofilm, antibiotic resistance, antibiotic tolerance, persister

## Abstract

In order to understand the role of biofilm in the emergence of antibiotic resistance, a total of 104 clinical *Acinetobacter baumannii* strains were investigated for their biofilm-forming capacities and genes associated with biofilm formation. Selected biofilm-formers were tested for antibiotic susceptibilities when grown in biofilm phase. Reversibility of antibiotic susceptibility in planktonic cells regrown from biofilm were investigated. We found 59.6% of the strains were biofilm-formers, among which, 66.1% were non-multidrug resistant (MDR) strains. Presence of virulence genes *bap*, *csuE*, and *abaI* was significantly associated with biofilm-forming capacities. When strains were grown in biofilm state, the minimum biofilm eradication concentrations were 44, 407, and 364 times higher than the minimum bactericidal concentrations (MBC) for colistin, ciprofloxacin, and imipenem, respectively. Persisters were detected after treating the biofilm at 32–256 times the MBC of planktonic cells. Reversibility test for antibiotic susceptibility showed that biofilm formation induced reversible antibiotic tolerance in the non-MDR strains but a higher level of irreversible resistance in the extensively drug-resistant (XDR) strain. In summary, we showed that the non-MDR strains were strong biofilm-formers. Presence of persisters in biofilm contributed to the reduced antibiotic susceptibilities. Biofilm-grown *Acinetobacter baumannii* has induced antibiotic tolerance in non-MDR strains and increased resistance levels in XDR strains. To address the regulatory mechanisms of biofilm-specific resistance, thorough investigations at genome and transcription levels are warranted.

## 1. Introduction

*A. baumannii* is a significant opportunistic pathogen responsible for a high proportion of healthcare-associated infections [1]. Due to the critical impact of the multidrug-resistant *A. baumannii* on public health, the World Health Organization has categorized this organism as a priority I pathogen among the antibiotic-resistant microorganisms [2,3,4,5,6]. Recent reports have shown that the environmental reservoir is the major source of multidrug-resistant *A. baumannii* outbreaks in hospital environments [7]. The ability of *A. baumannii* to form biofilm facilitates its survival and persistence in the hospital environments [8,9], this in turn contributes to the extensive spread of this pathogen across the globe [10]. 

Biofilm is a multilayer of highly coordinated microorganisms attached to surfaces in the presence of moisture. Bacterial cells within the biofilm are highly coordinated and undergo phenotype switch to produce a community that is resistant to the adverse external environment. Such phenotype switch also promotes the emergence of antibiotic resistance through the expression of the antibiotic-resistance genes, genetic mutation, or transfer of genes associated with antibiotic-resistance [11].

Virulence genes associated with biofilm formation in *A. baumannii* include *bap*, *csu* locus, *adeFGH*, *ompA*, and *abaI* [10]. *bap* encodes a large bacterial surface protein consisting of 8621 amino acids [11]. The predicted structure of Bap was similar to bacterial adhesins of the immunoglobulin-like fold superfamily and may function as an intercellular adhesin that supports the development of the mature biofilm structure [12]. *csuE* belongs to the *csu* operon, which encodes the chaperone-usher pili assembly system as production of pili is required in the early steps of biofilm formation on abiotic surfaces [13]. *ompA* encodes a porin protein that is involved in adhesion to the epithelial cell, antibiotic resistance, and biofilm formation [8]. *adeFGH* encodes a resistance–nodulation–cell division antibiotics efflux system, which is involved in the synthesis and transportation of autoinducer molecules during biofilm formation [14]. *abaI* encodes an autoinducer synthase, which is an enzyme involved in quorum sensing. Mutation of *abaI* fails to produce acyl-homoserine lactone signals and impairs biofilm maturation [5]. Certain biofilm-associated genes influence the expression of antibiotic resistance, suggesting the link between biofilm formation and antibiotic resistance.

In the past decade, most of the published studies on antimicrobial resistance of *A. baumannii* focused on the planktonic states of the pathogen [5,15,16]. However, biofilm of *A. baumannii* is responsible for various types of catheter-related infections, antimicrobial resistance performed using planktonic cells may not be representative for biofilms. In order to understand the virulence potential and the influence of biofilm growth on antibiotic susceptibility, we characterized the biofilm-producing capacities of the *A. baumannii* strains isolated from clinical specimens and to examine the responses of biofilm cells to antimicrobial agents. It has been documented that antibiotic-resistant strains of *A. baumannii* were strong biofilm formers [10], such properties are beneficial to the survival and dissemination of the resistant strains. However, antibiotic-sensitive strains are vulnerable to antibiotic challenges, in order to protect themselves from antibiotics present in the surrounding environments, the sensitive strains may have the abilities to form biofilm. Therefore, the study investigated the relationship between antibiotic susceptibility and biofilm-forming ability. The results obtained from the phenotypic characterization of the biofilm-producing capacity and biofilm susceptibility tests may offer valuable insights into the development of preventive strategies for biofilm-associated infections in hospital environments. 

## 2. Materials and Methods 

### 2.1. Clinical Isolates 

A total of 104 archived and nonduplicate *Acinetobacter species* isolates were collected from different hospitals in Hong Kong. All strains of *Acinetobacter* species were collected from sputum, blood, urine, soft tissue, and hospital environments. The collected isolates were kept in Luria-Bertani (Oxoid Ltd., Basingstoke, UK) broth containing 20% glycerol at −80 °C until use. The reference strains *A. baumannii* ATCC 19606 and *E. coli* ATCC 25922 were used as control strains in the biofilm assay or antibiotic susceptibility tests.

### 2.2. Confirmation of Bacterial Identities

Identities of the *A. baumannii* isolates were confirmed using a multiplex PCR assay. Three pairs of primers targeting *recA*, *gyrB*, and ITS were used [17]. PCR was performed in a final reaction volume of 40 μL containing 5X Phusion HF Buffer, 10 mM dNTP, and Phusion DNA Polymerase (all from New England BioLabs, MA, USA), and 0.5 µM of each primer using a Veriti thermocycler (Applied Biosystems, Foster City, CA, USA) under the following conditions: initial denaturation at 98 °C for 5 min; 35 cycles of denaturation (95 °C, 30 s), annealing (54 °C, 30 s) and extension (72 °C, 1 min); a final extension step at 72 °C for 10 min. PCR products were separated by 1.5% agarose gel electrophoresis the gel was stained with 0.5 μg/mL RedSafe nucleic acid staining solution (iNtRON biotechnology, Gyeonggi, Korea). The stained gel was visualized using a Gel Doc System XR (Bio-Rad Laboratories, Richmond, CA, USA).

### 2.3. Multilocus Sequence Typing (MLST)

The Oxford scheme of MLST targeting seven chromosomal housekeeping genes was performed according to the method described in the MLST database (http://pubmlst.org/abaumannii). The seven housekeeping genes (*gltA*, *gyrB*, *ghdB*, *recA*, *cpn60*, *gpi*, and *rpoD*) of 104 *A. baumannii* isolates were amplified and sequenced according to the method described by Bartual et al. [18]. The allele number of each gene was obtained by comparing its sequence with the reference sequences in the database. The sequence type of a given isolate was identified by matching the seven locus numbers obtained. If a sequence did not match with any reference sequences in the database, it was designated as a new allele. In addition, if the seven loci did not match with any existing allele combinations in the database, the isolates were regarded as new sequence types. 

### 2.4. Biofilm Formation 

Biofilm-forming capacities of the isolates were evaluated with a Calgary Biofilm Device (CBD) with slight modifications of the procedures described by Ceri et al. [19] and the manufacturer (Innovotech, Edmonton, AB, Canada). Briefly, 3–5 colonies were picked from an overnight LB agar plate and inoculated into 10 mL Maximum Recovery Diluent to a turbidity equivalent to 0.5 McFarland standard. The inoculum was mixed 1:1 with LB broth. For each *A. baumannii* strain, 150 μL of bacterial suspension was inoculated into the well of a 96-well microtiter plate. The plate was then covered with a plastic lid with 96 pegs (Innovotech). The microtiter plate was incubated at 37 °C for 48 h on a platform shaker set at 110 rpm. After incubation, planktonic cells were aspirated from the wells and discarded; the wells were washed three times with Maximum Recovery Diluent. Both the plates and lids with pegs were inverted and allowed to dry for 2 h at room temperature. Biofilm mass quantification was performed in octuplicate, and each assay was repeated on three separate days.

### 2.5. Quantification of Biofilm Mass

Biofilm masses formed on the 96-well microtiter plate was quantified using the crystal violet staining method. After drying of the microtiter plate and the lid with pegs, 200 μL of 0.1% aqueous crystal violet solution was added into each well of the microtiter plate, which was covered with the lid with pegs. The set up was incubated at room temperature for 15 min. After staining, the wells and the pegs were washed three times with Maximum Recovery Diluent to remove the excess stain and air-dried. After drying, 200 μL of 33% (*v*/*v*) acetic acid was added to each well of the microtiter plate to extract the crystal violet bound to the biofilm. Absorbance was measured at 570 nm using a microplate spectrophotometer (BIO-RAD). The average optical density (OD) for each *A. baumannii* isolate was calculated, and the biofilm-forming capacity was interpreted according to the guideline described by Stepanović et al. [20]. The cut-off OD (ODc) was defined as three standard deviations above the mean OD of the uninoculated control. Classification criteria are shown in Table 1. 

### 2.6. Detection of Biofilm-Associated Genes

PCR detection of biofilm-associated genes (*bap*, *csuE*, *ompA*, *adeFGH*, *abaI*) was performed on a Veriti thermocycler (Applied Biosystems) using sets of primers shown in Table S1. PCR was performed in a 20-µL reaction consisting of 1 µL extracted genomic DNA, 10 µL Luna Universal qPCR Master Mix (New England Biolabs, USA), 1 µL (10 µM) of each primer. The reaction condition was initial denaturation at 95 °C for 7 min, followed by 35 cycles of denaturation at 95 °C for 30 s, annealing at 60 °C for 1 min and extension at 72 °C for 30 s, and a final extension of 72 °C for 10 min. PCR products were separated by 1.5% agarose gel electrophoresis the gel was stained with 0.5 μg/mL RedSafe nucleic acid staining solution (iNtRON Biotechnology, Korea). The stained gel was visualized using a Gel Doc System XR (Bio-Rad Laboratories).

### 2.7. Determination of Minimal Inhibitory Concentration (MIC) and Minimum Bactericidal Concentration (MBC)

Antimicrobial susceptibility testing was determined by broth microdilution techniques according to the procedures described in the CLSI guidelines [21]. Interpretation of susceptible, intermediate, and resistant was based on CLSI guidelines. Ten antimicrobials covering seven categories of antimicrobials used for the treatment of *A. baumannii* infections were included in the determination of MIC and MBC. These included penicillin-β-lactamase inhibitor (ampicillin-sulbactam), cephalosporin (cefotaxime, ceftazidime), a carbapenem (imipenem, meropenem), aminoglycoside (gentamycin), a fluoroquinolone (ciprofloxacin, levofloxacin), tetracycline (tetracycline), lipopeptide (colistin) were included in the study. *E. coli* ATCC 25922 and *A. baumannii* ATCC 19606 (*A. baumannii* ST1861) were used as control strains. The strains were regarded as multidrug-resistant (MDR) if they were resistant to at least three classes of antimicrobial agents, including penicillin and cephalosporin (including inhibitor combinations), fluoroquinolones, and aminoglycosides. MDR strains that were resistant to carbapenem were regarded as extensively drug-resistant (XDR) [22]. XDR strains were resistant to colistin, and all other classes of antibiotics were regarded as pan drug-resistant (PDR). 

After MIC determination, 10 μL aliquots from the wells that showed no visible bacterial growth were inoculated onto LB agar plates and incubated at 37 °C for 24 h. The MBC was the lowest concentration of antibiotic that killed 99.9% of the initial bacterial population. 

### 2.8. Determination of Minimal Biofilm Inhibitory Concentration (MBIC) and Minimum Biofilm Eradication Concentration (MBEC) Assay 

The antibiotic susceptibility test for biofilm was performed according to the procedures described by Moskowitz et al. [23]. After the biofilm was formed on the CBD pegs, the pegs were rinsed three times in a 96-well plate containing 150 µL of Maximum Recovery Diluent. The lid with pegs was then transferred to the new a standard 96-well plate with wells containing 150 µL Mueller–Hinton broth with antibiotics diluted two-fold serially (ranged from 2 to 1024 µg/mL). The 96-well plate was incubated overnight at 37 °C. Following incubation, turbidity in each well was examined visually. MBIC was defined as the minimal antibiotic concentration at which no bacterial growth was observed, which meant the minimal antibiotic concentration that inhibited the release of planktonic bacteria from the biofilm. 

Following the MBIC examination, MBEC was determined. The lid with pegs was removed and rinsed three times in a 96-well plate containing 150 µL of Maximum Recovery Diluent to remove the planktonic cells. The lid was then placed in a second 96-well plate containing 150 µL Mueller–Hinton broth. The plate was shaken at a speed of 180 rpm for 10 min to detach the biofilm cells from the pegs into the Mueller–Hinton broth. The viability of the biofilm was determined by plate count after 24 h of incubation at 37 °C. MBEC was defined as the minimal antibiotic concentration required to eradicate the biofilm.

### 2.9. Confocal Laser Scanning Microscopy Imaging of Biofilm 

Confocal laser scanning microscopy (CLSM) was performed to estimate the proportion of viable cells in the biofilm formed on the pegs. After the formation of biofilm, the pegs were separated from the lid using sterilized pliers and washed three times with Maximum Recovery Diluent to remove the planktonic cells [19]. Biofilm cells on the pegs were dual stained with a mixture of 3.35 µM SYTO-9 and 20 µM propidium iodide according to the instructions of the Film Tracer Live/Dead Biofilm Viability Kit (Cat no: L10316 Invitrogen). CLSM images were acquired using a Leica TCS SPE Confocal Microscope (Leica) with a 63x objective lens. The live and dead cells embedded in subpopulations of biofilm cells were estimated using a software BioFilmAnalyzer [24].

## 3. Reversibility of Antibiotic Resistance of the Biofilm Cells

A study was performed to evaluate the antibiotic susceptibility profile after the biofilm cells were grown in the planktonic state. Two hyper-biofilm forming *A. baumannii* (ST1894 and ST373) and one weak biofilm former (ST195) were selected for this part of the study. ST1894 and ST373 were non-MDR strains; ST195 was an XDR strain. The biofilm cells of these three *A. baumannii* strains were subcultured on LB agar. MBICs of three antibiotics (colistin, imipenem, and ciprofloxacin) for the biofilm cells and MICs for the resumed planktonic cells were determined according to the procedures described above.

### 3.1. Enumeration of Persister Cells from Planktonic and Biofilm Populations

Persister cells in the planktonic and biofilm populations were enumerated according to the procedures described by Marques [25] with slight modification. A hyper-biofilm-producing and non-MDR *A. baumannii* ST1894 strain were selected for the isolation of persister cells. The strain was streaked onto a LB agar plate and incubated at 37 °C for 24 h. One to two colonies were inoculated into 5 mL LB broth, which was incubated at 37 °C with agitation (180 rpm) for 12 h. After incubation, the broth culture was diluted to 1% in 20 mL of fresh LB broth and incubated at 37 °C with agitation (180 rpm) for 24 h. 

To enumerate persister cells in the planktonic population, 10 mL of overnight LB broth culture of ST1894 was collected by centrifugation at 8000 rpm for 10 min at 4 °C and resuspended in 10 mL of saline at 4 °C. The washing step was repeated one more time. Cell density was adjusted to an absorbance of 0.8 at 600 nm. A duplicated set of bacterial cell suspension was prepared in the same way. Ninty-eight microliters of 2048 µg/mL ciprofloxacin (20× MIC) or 98 µL saline containing 0.1% acetic acid was added to the 10 mL bacterial cell suspension and incubated at 37 °C with agitation at 180 rpm for 24 h. Viable bacterial cells were enumerated using the plate count method. One hundred microliters of the experiment and control samples were inoculated onto the LB agar plate containing 1% MgCl_2_.7H_2_O to neutralize the ciprofloxacin. Plate count was performed in triplicate at time points 0, 1, 3, 5, 7, 9, 12, 15, and 24 h after incubation. The number of persister cells present in the planktonic population was determined according to the formula shown below:

$$\mathrm{The}\;\mathrm{number}\;\mathrm{of}\;\mathrm{persisters}\;\mathrm{in}\;\mathrm{planktonic}\;\mathrm{cells}/\mathrm{mL}\;=\frac{\mathrm{Number}\;\mathrm{of}\;\mathrm{colonies}\times \mathrm{dilution}\;\mathrm{factor}}{\mathrm{Volume}\;\mathrm{plated}}$$

To enumerate persister cells in the biofilm population, an overnight LB broth culture of ST1894 was used to grow biofilm using a CBD according to the procedures described elsewhere in this article. After incubation for 48 h, the pegs were removed from the CBD using pliers and each peg was placed in a centrifuge tube with 1 mL Maximum Recovery Diluent. The biofilm cells formed on the pegs were detached by centrifuging at 18,000 rpm for 10 min and were resuspended in 10 mL saline at 4 °C. Cell density was adjusted to an absorbance of 0.8 at 600 nm. Ninty-eight microliters of 2048 µg/mL ciprofloxacin (20 × MIC) or 98 µL saline containing 0.1% acetic acid was added to the 10 mL bacterial cell suspension and incubated at 37 °C with agitation at 180 rpm for 24 h. The number of viable biofilm cells for each treated and nontreated sample was counted according to the steps as described for planktonic cells. The number of persister cells isolated from the biofilm population was determined according to the formula shown below:

$$\mathrm{Number}\;\mathrm{of}\;\mathrm{persisters}\;\mathrm{in}\;\mathrm{biofilm}/\mathrm{mL}\;=\frac{\mathrm{Number}\;\mathrm{of}\;\mathrm{colonies}\times \mathrm{dilution}\;\mathrm{factor}\times \mathrm{total}\;\mathrm{sample}\;\mathrm{volume}}{\mathrm{Volume}\;\mathrm{plated}\times \mathrm{surface}\;\mathrm{area}\;\mathrm{of}\;\mathrm{peg}}$$

### 3.2. Statistical Analysis

The association between biofilm-forming abilities and biofilm-associated genes was analyzed using chi-squared tests. SPSS version 24 (IBM Corporation, Armonk, NY, USA) was used for statistical analysis. *p*-value ≤0.05 was considered as statistically significant for the data analyzed in this study. 

## 4. Results

### 4.1. Antibiotic Susceptibility Profiles of the A. baumannii Strains 

The MICs for 10 antibiotics (listed in Table 2) covering seven major antibiotic classes were determined for the 104 *A. baumannii* isolates. The antibiotic susceptibility profiles of the isolates are summarized in Table 2. The results showed that 79.8% (82/104) of the isolates were susceptible to colistin, but only 4.8% (5/104) of the isolates were susceptible to ampicillin-sulbactam. There were 29.8% (31/104) to 52.9% (55/104) of isolates susceptible to the remaining eight antibiotics. Among the 104 isolates, 46.2% (48/104) of strains were non-MDR strains, 30.8% (32/104) were XDR strains, and 23.1% (24/104) were PDR strains (Table 3 and Figure S1).

### 4.2. Biofilm-Forming Capacities of the A. baumannii Isolates

Among the 104 *A. baumannii* isolates studied, 59.6% were biofilm formers, of which 25% were strong biofilm producers, 14.4% were moderate biofilm producers, and 20.2% were weak biofilm. The biofilm control strain *A. baumannii* ATCC19606 was a strong biofilm former. Table 3 summarizes the distribution of biofilm-forming abilities of isolates with various antibiotic susceptibility and biofilm-associated gene profiles. As shown in Table 3, the result reveals that 66.1% of the biofilm-forming isolates were non-MDR strains. In addition, 83.3% of the non-biofilm formers were resistant to multiple antibiotic classes. Overall, these results indicated that the more antibiotic susceptible *A. baumannii* isolates were able to form biofilm than the resistant isolates (*p* = 3 × 10^−6^). 

The biofilm-forming capacities of each sequence type are also summarized in Figure S1. The results showed that the isolates with the same sequence type had different biofilm-producing capacities, as in the case of ST2028, ST1417, ST195, ST1860, and ST2037.

### 4.3. Distribution of Biofilm-Associated Virulence Genes in the Clinical A. baumannii Strains

The percentages of *A. baumannii* strains carrying *bap*, *csuE*, *adeFGH*, *ompA*, and *abaI* were 9.6%, 85.6%, 95.2%, 89.4%, and 82.7%, respectively. The relationship between the presence of biofilm-associated genes and biofilm-forming capacities are presented in Table 4. The presence of *bap*, *csuE*, and *abaI* was significantly associated with biofilm-forming capacities of the isolates (*p*-values ranging from 0.005 to 0.033). The results showed that 100% of *bap*-positive isolates were biofilm formers. On the other hand, 55.3% of biofilm formers were *bap*-negative. Based on the results, the presence of *Bap* indicates the strains are biofilm-formers but the reverse is not true. Over 80% of the *csuE*-negative and *abaI*-negative isolates were biofilm formers, but more than 50% biofilm formers carried these two genes. Similar patterns were observed in *adeFGH* and *ompA* but were not statistically significant (*p*-values = 0.07 and 0.193, respectively).

### 4.4. Comparison of MIC and MBIC

The MICs and MBICs for colistin, ciprofloxacin, and imipenem were tested against nine different sequence types. These strains were biofilm-forming and susceptible to the three antibiotics being investigated. Antibiotic resistance profiles for biofilm compared to counter-plankton cells are summarized in Table 5. The MBICs increased drastically when compared to those of the planktonic cells. The fold-increase ranged from 2 to 32 fold for colistin, 4 to 64 fold for ciprofloxacin, and 4 to 2048 fold for imipenem. When compared between MICs and MBICs, the average fold-increase was 21, 31, and 386 for colistin, ciprofloxacin, and imipenem, respectively. The susceptibility profiles of most of the strains changed from sensitive to resistant when grown in biofilm state. However, the biofilm formed by ST1990 and ST1417 remained sensitive to ciprofloxacin and imipenem, and biofilm formed by ST1855 remained sensitive to colistin.

### 4.5. Comparison of MBC and MBEC

**Figure 1:**
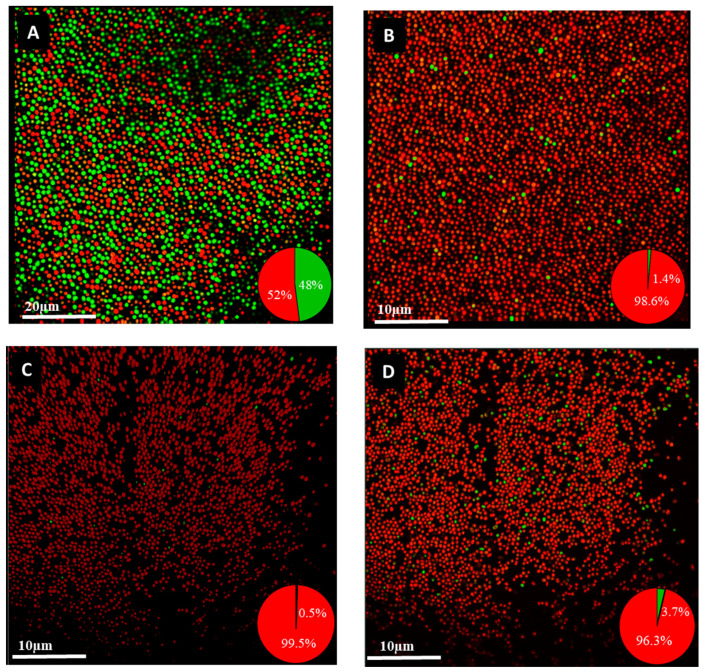
CLSM images of *A. baumannii* ST1894 biofilm treated with bactericidal antibiotics. (**A**) Untreated biofilm cells, (**B**) biofilm treated with 512 µg/mL imipenem, (**C**) biofilm treated with 128 µg/mL colistin, (**D**) biofilm treated with 512 µg/mL ciprofloxacin. Biofilm was incubated with antibiotics at 37 °C for 48 h, which was followed by costaining with propidium iodide (PI) and SYTO 9, and examined under CLSM. Dead cells were stained with PI and appeared red. Viable cells were stained with SYTO 9 and appeared green in color.

Following the determination of MICs and MBICs, we evaluated the MBCs and MBECs of nine *A. baumannii* isolates. The results showed that the MBECs of the nine isolates have increased. Up to 64-fold increase in the concentration of colistin and up to a 1024-fold increase in the concentration of ciprofloxacin and imipenem was required to eradicate *A. baumannii* biofilm compared to the planktonic cells (Table 6). Although the three strains, ST1990, ST1417, and ST1855, had MBICs falling within the sensitive ranges, the MBECs were much higher than the MBICs (Table 5). When compared between MBCs and MBECs, the average fold-increase was 44, 407, and 364 for colistin, ciprofloxacin, and imipenem, respectively. To visualize the viability of biofilm cells after antibiotic treatment, biofilm preparations of a hyper-biofilm-producing strain ST1894 were treated with antibiotics, stained with a LIVE/DEAD BacLight bacterial viability kit and examined using CLSM. The results showed that viable cells in the biofilm can still be detected even after treating with antibiotics at 32–256 times the MBC (Figure 1). The findings of this study suggest that biofilm-producing strains *A. baumannii* cannot be eradicated with the same concentration of antimicrobials required to eradicate planktonic cells.

### 4.6. Reversibility of Antibiotic Susceptibility in Planktonic Cells Regrown from Biofilm 

Reversibility of antibiotic resistance was analyzed for two non-MDR strains *A. baumannii* ST1894, ST373, and one XDR strain *A. baumannii* ST195. Biofilm cells of the three strains were regrown into the planktonic phase and then treated with colistin, imipenem, and ciprofloxacin. The MIC, MBIC, and MIC of the reverted planktonic cells are summarized in Table 7. The results showed that biofilm cells of *A. baumannii* ST1894 reverted to sensitive phenotype when the strain was regrown into planktonic cells. For *A. baumannii* ST373, reversion to sensitive phenotype occurred in colistin and imipenem but not in ciprofloxacin. This suggests that biofilm cells of that strain might have developed mutation associated with ciprofloxacin resistance. Besides, other mechanisms might be involved, such as mutations in the efflux pumps or regulators of efflux pumps could also lead to ciprofloxacin resistance. *A. baumannii* ST195 was resistant to ciprofloxacin and imipenem but sensitive to colistin. The MICs of the reverted planktonic cells were the same as the MBICs for the three antibiotics. The results showed that the antibiotic susceptibility of the strain did not revert to the original pattern. The level of resistance to ciprofloxacin and imipenem has increased, and the strain did not revert to its sensitive phenotype for colistin. Together the results showed that biofilm formation in *A. baumannii* promotes either antibiotic tolerance that is reversible or the emergence of antibiotics resistance that is irreversible.

### 4.7. Isolation of Persister Cells in Planktonic and Biofilm Cells 

**Figure 2:**
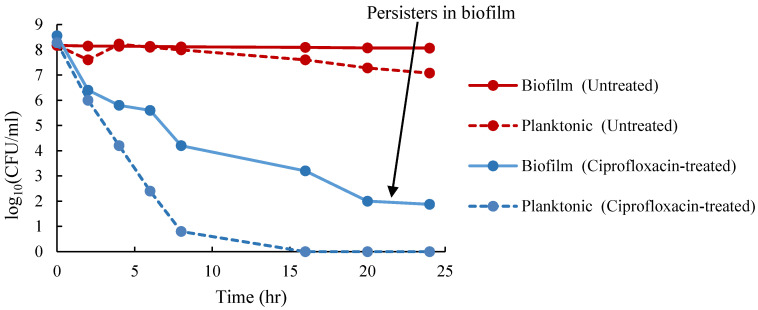
Detection of persisters from biofilm and planktonic cells of *A. baumannii* ST 1894. The number of viable biofilm and planktonic cells at different time points after treatment with 2048 μg/mL ciprofloxacin. Red solid and dotted lines represent untreated biofilm and planktonic cells, respectively. Blue solid and dotted lines represent biofilm and planktonic cells treated with ciprofloxacin, respectively.

**Figure 3:**
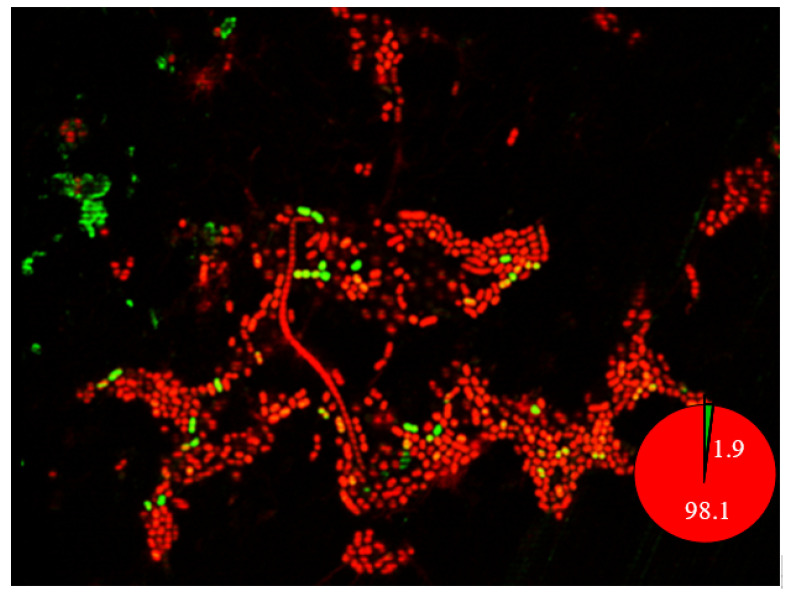
CLSM image of *A. baumannii* ST1894 biofilm treated with ciprofloxacin at 1024 × MIC. Viable bacterial cells in the biofilm was detected by a Live/Dead Biofilm Viability Kit. Persister cells appear green in color, dead cells appear red in color.

When *A. baumannii* ST1894 was treated with ciprofloxacin at a concentration equaled 20 times of its MIC, all planktonic cells were eradicated after 16 h of exposure to the antibiotic (Figure 2). However, 100 ± 30 CFU/peg biofilm cells survived after 24-h exposure to ciprofloxacin, which supports our hypothesis that persister cells in the biofilm was one of the reasons for the reduced susceptibility to antibiotics. Besides, these persister cells were responsible for the regrowth of biofilm cells after the cells were treated with antibiotic. Further examination of biofilm using CLSM demonstrated the presence of 1.9% persister cells when the biofilm was treated with ciprofloxacin at concentration of 1024 times of its MIC (Figure 3). Hence, the presence of persister cells in biofilm could be the reason for multidrug tolerance to different classes of antibiotics.

## 5. Discussion

The ability of *A. baumannii* to produce biofilm enhances its survival in adverse environments and increases the risk of healthcare-associated infections. The present study evaluated the biofilm-formation abilities of the clinical *A. baumannii* strains and the role of biofilm production for reduced susceptibility to antibiotics. The result showed that 59.6% of the clinical *A. baumannii* isolates were able to form a biofilm. This result is similar to the findings reported by Anghel et al. that about 63% of *A. baumannii* clinical strains were biofilm producers [26]. Our results showed that the strains with the same sequence types might have different biofilm-producing capacities, as in the case of ST2028, ST1417, ST195, ST1860, and ST2037.

Previous studies have documented that biofilm-forming strains were more resistant to antibiotics than the non-biofilm-forming strains [10,27]. However, in this study, we found that a significantly higher proportion of biofilm-forming isolates were non-MDR strains. Our results indicated an inverse relation between antibiotic resistance and biofilm-forming ability of the isolates. This finding is also in line with other studies that biofilm-forming *A. baumannii* was more susceptible to antibiotics [28,29]. A possible explanation for this might be that biofilm formation ensures the survival of the susceptible strains when exposed to antibiotics. However, the variations in methodologies involved in assessing biofilm formation may lead to different observations among different research groups. A standardized biofilm assay is needed for more objective comparison between different studies.

In this study, we observed that the majority of XDR and PDR *A. baumannii* isolates were weak or non-biofilm producers. The results are in accordance with the recent findings that biofilm-forming isolates exhibited lower rates of carbapenem resistance than the non-biofilm-forming isolates [30,31]. This result may be explained by the fact that energy required for expressing the carbapenemase and β-lactamases might reduce the biofilm formation in the isolates harboring those genes. 

We also evaluated the relationship between biofilm-associated virulence genes and biofilm-forming abilities of the *A. baumannii* strains. *bap* was detected in a small proportion of strains tested, and all *bap*-positive strains were biofilm-formers, indicating the role of *Bap* in biofilm formation. It has been documented that *Bap* protein is necessary for mature biofilm formation on various abiotic surfaces; disruption of *Bap* resulted in a reduction of biofilm mass [32]. *csuE, adeFGH*, *ompA*, and *abaI* were detected in 82.7–95.2% of the *A. baumannii* strains; almost 50% of these strains were non-biofilm-forming strains. It is, therefore, necessary to compare the expression levels of the biofilm-associated genes in the biofilm producers and non-biofilm producers. 

To examine the emergence of antibiotic resistance during biofilm formation, MIC, MBIC, MBC, and MBEC of colistin, ciprofloxacin, and imipenem against nine selected *A. baumannii* strains were studied. These nine strains were non-MDR and strong biofilm formers. The results showed that biofilm cells of the nine strains developed a high-level of antibiotic resistance and required as high as 2048 times the MIC to inhibit the release of planktonic bacterial cells from biofilm or as high as 1024 times the MBC to eradicate the biofilm cells. Among the three antibiotics tested, imipenem had a high average fold-increase in both MBIC (386-fold) and MBEC (364-fold). As imipenem is uncharged and can penetrate the negatively charged biofilm matrix, thus, the high level of imipenem resistance was unlikely due to poor penetration. Resistance could be a result of the expression of β-lactamase, as a previous study reported that the activity of the β-lactamase promoter was elevated in biofilm cells of *Pseudomonas aeruginosa* [33]. Other possibilities for the emergence of imipenem resistance in the biofilm include the expression of the drug efflux system *adeFGH*, which is involved in the synthesis and transportation of autoinducer molecules during biofilm formation [14]. 

Ciprofloxacin had much higher fold-increase in MBEC than that in MBIC (407-fold vs. 31-fold), as ciprofloxacin is actively against metabolically active bacterial cells [34], it will be more difficult to eradicate persister cells developed in the biofilm, which are metabolically dormant. Colistin had the lowest average fold-increase in MBIC (21-fold) and MBEC (44-fold) compared to imipenem and ciprofloxacin. Studies also showed that colistin is effective against the metabolically inactive bacterial population in the biofilm [34]. Another study also showed that the bactericidal activity of colistin increased under anaerobic conditions, as the bactericidal action of the antibiotic was independent of the hydroxyl radical formed during aerobic respiration [34]. Overall, *A. baumannii* developed antibiotic resistance during biofilm formation, and the level of biofilm-specific resistance varied according to the induced responses of the biofilm population and the action mechanisms of the antibiotics.

CLSM images of a non-MDR *A. baumannii* ST1984 strain showed that 0.5%–3.7% of biofilm cells were viable after treatment with colistin, ciprofloxacin, and imipenem at 256–4096 times the MICs of these antibiotics. It is possible that the percentages of viable cells were higher because the presence of extracellular DNA in the biofilm might be stained by the propidium iodide stain and lead to an overestimation of dead cell proportions. The presence of persister cells is worrisome since colistin is the last resort for treatment of MDR *A. baumannii*, the emergence of biofilm-specific resistance makes the pathogen untreatable with the conventional antibiotics. 

Although Qi et al. have reported the enhancement of antibiotic resistance by biofilm formation [28], our study investigated the reversibility of antibiotic resistance developed in the biofilm. We re-grew the biofilm cells to planktonic state and assessed the changes in MICs in three *A. baumannii* strains. Our novel findings demonstrated that reversion from resistant to susceptible occurred in two non-MDR strains but not in the XDR strain. Such a transient increase in antibiotic resistance in the non-MDR strains was due to the tolerance of antibiotics by the biofilm cells. There are multiple mechanisms involved in the development of antibiotic tolerance in biofilm. The reduced metabolic activity of biofilm and induction of stress responses due to the limitation of nutrients in the biofilm environment triggers antibiotic tolerance [35]. The presence of persister cells in the biofilm population activates the toxin/antitoxin systems, which leads to inhibition of protein translation, initiation of dormancy, and tolerance to antibiotics [36]. In this study, we demonstrated the presence of persister cells in *A. baumannii* strain ST1894, one of the two non-MDR strains, using CLSM imaging and persister cell isolation after treating with a high concentration of ciprofloxacin. The presence of persister cells made the pathogen extremely multidrug tolerant and could eventually lead to the emergence of antibiotic resistance. For the XDR strain (*A. baumannii* ST195), the planktonic cells regrown from biofilm cells exhibited a higher level of antibiotic resistance. This could be caused by mutations of drug targets upon exposure to high antibiotic concentrations. There are two noteworthy issues regarding this XDR strain. First, irreversible colistin resistance was developed during biofilm formation, making the physicians barehanded to treat the infected patients. Second, although the strain was a weak biofilm former, high-level of irreversible antibiotic resistance was enhanced during biofilm formation. Detailed investigation at the genetic level is needed to understand the underlying regulatory mechanisms involved in the emergence of biofilm-mediated antibiotic resistance. 

## 6. Conclusions

We showed a negative relationship between biofilm-forming capacity and antibiotic susceptibility, in which the strong biofilm-formers were non-MDR strains. We also demonstrated the presence of persisters in the biofilm cells, which accounted for reduced susceptibilities of the *A. baumannii* strains. Another important finding was that growth in biofilm induced reversible antibiotic tolerance in the non-MDR strains but a higher level of irreversible resistance in the XDR strain, including the conversion from colistin sensitive to resistant. To address the regulatory mechanisms of biofilm-specific resistance, research should be undertaken to investigate the alterations at the genome and transcription levels of *A. baumannii* grown in biofilm status. A thorough understanding of the various contributing factors facilitates the design of new therapeutics that target biofilm-specific resistance.

## Figures and Tables

**Table 1:** Classification of biofilm-forming capacity based on optical density (OD) value.

Classification	OD Range		
Non-biofilm producer		OD	≤ODc
Weak biofilm producer	ODc	<OD	≤2 × ODc
Moderate biofilm producer	2 × ODc	<OD	≤4 × ODc
Strong biofilm producer		OD	>4 × ODc

**Table 2:** Antimicrobials susceptibility profiles the *Acinetobacter baumannii* isolates (n=104). S, I, and R represent susceptible, intermediate, and resistant, respectively.

Antimicrobials	MIC Breakpoints (µg/mL)			Susceptible (%)	Intermediate (%)	Resistant (%)
	Susceptible	Intermediate	Resistant			
Colistin	≤2	**-**	≥4	79.8	-	20.2
Imipenem	≤2	4	≥8	48.1	-	51.9
Meropenem	≤2	4	≥8	48.1	-	51.9
Cefotaxime	≤8	16–32	≥64	29.8	13.5	56.7
Ceftazidime	≤8	16	≥32	39.4	-	60.6
Ciprofloxacin	≤1	2	≥4	48.1	-	51.9
Levofloxacin	≤1	2	≥4	48.1	-	51.9
Gentamycin	≤2	4	≥8	52.9	-	47.1
Tetracycline	≤4	8	≥16	48.1	-	51.9
Ampicillin-sulbactam	≤4	8	≥16	4.8	3.8	91.3

**Table 3:** The relationship among biofilm-producing ability of *A. baumannii* with antibiotic resistance.

Biofilm-Producing Ability	% of Isolates	% of Isolates with Different Antibiotic Susceptibility Profiles		
		Non-MDR 46.2% (48/104)	XDR 30.8% (32/104)	PDR 23.1% (24/104)
Biofilm-forming	>59.6% (62/104)	>66.1% (41/62)	>17.7% (11/62)	>16.1% (10/62)
Strong	25% (26/104)	92.3% (24/26)	7.7% (2/26)	0% (0/26)
Moderate	14.4% (15/104)	66.7% (10/15)	26.7% (4/15)	6.7% (1/15)
Weak	20.2% (21/104)	33.3% (7/21)	23.8% (5/21)	42.9% (9/21)
Non-biofilm-forming	40.4% (42/104)	16.7% (7/42)	50% (21/42)	33.3% (14/42)

**Table 4:** Relationship between the presence of biofilm-associated genes and biofilm-forming capacities among the *A. baumannii* isolates.

Biofilm-Associated Gene	Biofilm-Forming No. (%)	Non-Biofilm-Forming No. (%)	*p*-Value
*bap*			0.005
Positive	10 (100)	0 (0)	
Negative	52 (55.3)	42 (44.7)	
*csuE*			0.02
Positive	49 (55.1)	40 (44.9)	
Negative	13 (86.7)	2 (13.3)	
*adeFGH*			0.07
Positive	57 (57.6)	42 (42.4)	
Negative	5 (100.0)	0 (0)	
*ompA*			0.193
Positive	53 (57)	40 (43)	
Negative	9 (81.8)	2 (18.2)	
*abaI*			0.033
Positive	47 (54.7)	39 (45.3)	
Negative	15 (83.3)	3 (16.7)	

**Table 5:** Antibiotic susceptibility profiles of selected sequence types of *A. baumannii* planktonic and biofilm cells.

	Colistin			Ciprofloxacin			Imipenem		
MLST	MIC for Planktonic Cells (µg/mL)	MBIC for Biofilm Cells (µg/mL)	Fold Change	MIC for planktonic Cells (µg/mL)	MBIC for Biofilm Cells (µg/mL)	Fold Change	MIC for Planktonic Cells (µg/mL)	MBIC for Biofilm Cells (µg/mL)	Fold Change
ST1894	0.5	16	32	1	64	64	0.125	256	2048
ST1990	0.5	8	16	0.5	2	4	0.125	2	16
ST1417	0.25	8	32	0.5	2	4	0.25	4	16
ST1992	2	4	2	0.5	4	8	0.25	32	128
ST373	1	32	32	0.5	16	32	0.25	32	128
ST1862	1	4	4	1	8	8	0.015	8	533.3
ST1964	0.5	16	32	1	64	64	4	16	4
ST1855	0.5	2	4	0.5	16	32	0.015	8	533.3
ST1861 *	0.5	16	32	1	64	64	0.5	32	64

* This strain is *Acinetobacter baumannii* ATCC 19606, the control strain for biofilm assay.

**Table 6:** Comparison of MBC and MBEC of *A. baumannii* of different MLSTs.

	Colistin			Ciprofloxacin			Imipenem		
MLST	MBC for Planktonic Cells (µg/mL)	MBEC for Biofilm Cells (µg/mL)	Fold Change	MBC for Planktonic Cells (µg/mL)	MBEC for Biofilm Cells (µg/mL)	Fold Change	MBC for Planktonic Cells (µg/mL)	MBEC for Biofilm Cells (µg/mL)	Fold Change
ST1894	4	256	64	8	1024	128	4	1024	256
ST1990	0.5	32	64	1	1024	1024	1	64	64
ST1417	1	32	32	2	64	32	0.5	64	128
ST1992	2	8	4	1	1024	1024	1	1024	1024
ST373	4	128	32	32	1024	32	4	32	8
ST1862	2	128	64	2	512	256	1	1024	1024
ST1964	4	256	64	2	32	16	4	512	128
ST1855	0.5	32	64	1	1024	1024	2	1024	512
ST1861 *	4	16	4	8	1024	128	1	128	128

* This strain is *Acinetobacter baumannii* ATCC 19606, the control strain for biofilm assay.

**Table 7:** Reversibility of antibiotic resistance of biofilm cells.

Strain	Biofilm Forming	Colistin			Ciprofloxacin			Imipenem			Reason for Reduced Susceptibility in Biofilm
		MIC	MBIC	MIC of Reverted Planktonic Cells	MIC	MBIC	MIC of Reverted Planktonic Cells	MIC	MBIC	MIC of Reverted Planktonic Cells	
ST1894 Non-MDR	Strong	0.5	16	0.5	1	64	1	0.125	256	0.125	Tolerance
ST373 Non-MDR	Strong	1	32	1	0.5	16	16	0.25	32	0.25	Tolerance or resistant mutant for ciprofloxacin
ST195 XDR	Weak	1	16	16	4	64	64	8	32	32	Resistant mutant

## Supplementary Materials

The following are available online at https://www.mdpi.com/2079-6382/9/11/817/s1, Figure S1: Biofilm-producing abilities and antibiotic susceptibility profiles of *A. baumannii* strains with different MLSTs, Table S1: Primers used for amplification of biofilm-associated genes.
