# Supplementary material for: Biofilm-Induced Antibiotic Resistance in Clinical Acinetobacter baumannii Isolates
Source: Antibiotics (Basel). 2020 Nov 17;9(11):817. doi: 10.3390/antibiotics9110817 (PMC7698371; doi:10.3390/antibiotics9110817)
Supplement: Supplementary file 1 [file antibiotics-09-00817-s001.zip › Supplementary files/Figure S1.pdf]

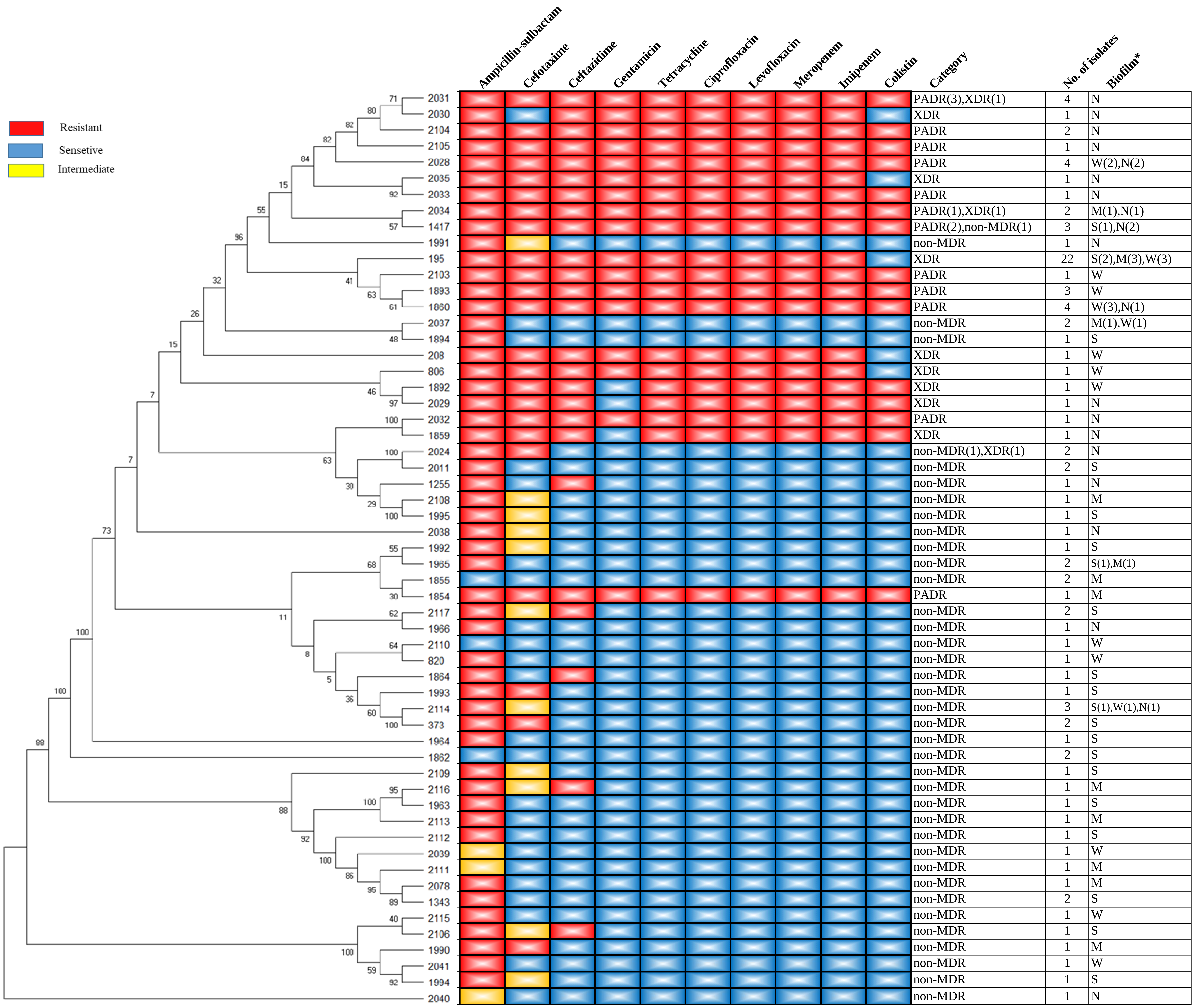

Figure S1. Biofilm-producing abilities and antibiotic susceptibility profiles of *A. baumannii* strains with different MLSTs.

* N: non-biofilm producer; W: weak biofilm-producer; M: moderate biofilm-producer; S: strong biofilm-producer. The bracketed number is the number of strains belongs to a particular bioflm-producing type.
