# Supplementary material for: Biofilm-Induced Antibiotic Resistance in Clinical Acinetobacter baumannii Isolates
Source: Antibiotics (Basel). 2020 Nov 17;9(11):817. doi: 10.3390/antibiotics9110817 (PMC7698371; doi:10.3390/antibiotics9110817)
Supplement: Supplementary file 1 [file antibiotics-09-00817-s001.zip › Supplementary files/Table S1.pdf]

Table S1. Primers used for amplification of biofilm-associated genes.

Target gene	Primers (5′- 3′)	Amplicon size (bp)	Annealing Temperature	Reference
bap	GGTACAAACTATGTGCCGGATT<br>CTGTATTCACTCCTTGACCAGC	934	60	1
csuE	AGACATGAGTAGCTTTACG<br>CTTCCCCATCGGTCATTC	516	60	2
ompA	CTGGTGTTGGTGCTTTCTGG<br>GTGTGACCTTCGATACGTG	352	60	2
adeFGH	TTCATCTAGCCAAGCAGAAG<br>CCTGCTAATGGTAGGGTTAAG	201	60	3
abaI	CCACACAACCCTATTTACTCGG<br>GGCGGTTTTGAAAAATCTACGG	121	60	1

References

1. Farshadzadeh Z, Taheri B, Rahimi S, Shoja S, Pourhajibagher M, Haghighi MA, Bahador A. Growth rate and biofilm formation ability of clinical and laboratory-evolved colistin-resistant strains of *Acinetobacter baumannii*. *Front. Microbiol.* **2018**, 9, doi.org/10.3389/fmicb.2018.00153

2. Ghasemi E, Ghalavand Z, Goudarzi H, Yeganeh F, Hashemi A, Dabiri H, Mirsamadi ES, Foroumand M. Phenotypic and genotypic investigation of biofilm formation in clinical and environmental isolates of *Acinetobacter baumannii*. *Arch. Clin. Infect. Dis.* **2018**, 13, e12914, doi: 10.5812/archcid.12914.

3. Yoon EJ, Courvalin P, Grillot-Courvalin C. RND-type efflux pumps in multidrug-resistant clinical isolates of *Acinetobacter baumannii*: Major role for AdeABC overexpression and AdeRS mutations. *Antimicrob. Agents. Chemother.* **2013**, 57, 2989–95.
